# In Vitro Study of Amniotic Fluid Gram Stain: Effect of Centrifugation

**DOI:** 10.1155/S1064744994000256

**Published:** 1994

**Authors:** Daniel W. Gauthier, Wilfredo Torres, William J. Meyer, Barbara G. Lewis, Michael O. Vernon, William M. Janda

**Affiliations:** 1 Department of Obstetrics and Gynecology Division of Maternal-Fetal Medicine University of Illinois College of Medicine 820 South Wood Street, M/C 808, Room 250 Chicago IL 60612-7313 USA; 2 Department of Pathology University of Illinois College of Medicine Chicago IL USA

## Abstract

*Objective:* Gram stain of amniotic fluid (AF) is used to detect intraamniotic infection. The purpose of this study was to determine if centrifugation improved the ability of AF Gram stain to detect bacteria.

*Methods:* AF obtained by amniocentesis from patients with preterm labor (PTL) or preterm premature rupture of membranes (PPROM) was pooled. Individual AF samples as well as the pooled sample had a negative Gram stain for microorganisms or white blood cells (WBCs) and negative cultures. With pure bacterial cultures, a suspension equivalent to a 0.5 McFarland turbidity standard was prepared and then serially diluted in the AF to either 10^6^, 10^5^, 10^4^, or 10^3^ colony forming units (cfu)/ml. Each sample was divided into 2 equal portions, with 1 undergoing centrifugation. The Gram stains were interpreted by technologists in the clinical microbiology laboratory in a blinded fashion. Fisher's exact test was used to compare the bacterial detection rate in centrifuged vs. uncentrifuged AF samples at each concentration.

*Results:* Centrifugation of AF significantly improved the ability of the Gram stain to detect bacteria at bacterial concentrations ≤10^4^ cfu/ml (*P* < 0.01). At concentrations ≥10^5^ cfu/ml, centrifugation did not improve the ability of the Gram stain to dtect bacteria.

*Conclusions: * At low bacterial concentrations, centrifugation of AF increases the bacterial detection rate of AF Gram stain.


Infectious Diseases in Obstetrics and Gynecology 1:282–284 (1994)
© 1994 Wiley-Liss, Inc.

### In Vitro Study of Amniotic Fluid Gram Stain: Effect of Centrifugation

Daniel W. Gauthier, Wilfredo Torres, William J. Meyer, Barbara G. Lewis, Michael O. Vernon, and William M. Janda

*Department of Obstetrics and Gynecology (D.W.G., W.T., W.J.M.), Division of Maternal-Fetal Medicine, and Department of Pathology (B.G.L., M.O.V., W.M.J.), University of Illinois College of Medicine, Chicago, IL*

ABSTRACT

***Objective:*** **Gram stain of amniotic fluid (AF) is used to detect intraamniotic infection. The purpose of this study was to determine if centrifugation improved the ability of AF Gram stain to detect bacteria.**

***Methods:*** **AF obtained by amniocentesis from patients with preterm labor (PTL) or preterm premature rupture of membranes (PPROM) was pooled. Individual AF samples as well as the pooled sample had a negative Gram stain for microorganisms or white blood cells (WBCs) and negative cultures. With pure bacterial cultures, a suspension equivalent to a 0.5 McFarland turbidity standard was prepared and then serially diluted in the AF to either $10^6$, $10^5$, $10^4$, or $10^3$ colony forming units (cfu)/ml. Each sample was divided into 2 equal portions, with 1 undergoing centrifugation. The Gram stains were interpreted by technologists in the clinical microbiology laboratory in a blinded fashion. Fisher's exact test was used to compare the bacterial detection rate in centrifuged vs. uncentrifuged AF samples at each concentration.**

***Results:*** **Centrifugation of AF significantly improved the ability of the Gram stain to detect bacteria at bacterial concentrations $\leq 10^4$ cfu/ml ($P < 0.01$). At concentrations $\geq 10^5$ cfu/ml, centrifugation did not improve the ability of the Gram stain to detect bacteria.**

***Conclusions:*** **At low bacterial concentrations, centrifugation of AF increases the bacterial detection rate of AF Gram stain.** © 1994 Wiley-Liss, Inc.

KEY WORDS

Intraamniotic infection, detection rate, rapid test

Amniotic fluid (AF) culture has been used to detect subclinical intraamniotic infection. A major drawback of using AF cultures for the detection of early infection is that results may not be available for 2 or more days. In centers where amniocentesis is performed to detect infection, antepartum management is often based on Gram stain results.

The disadvantage of using the Gram stain as a rapid predictor of AF culture results is a low sensitivity, ranging from 39% to 83%.[1–4] Romero et al.[4] reported that centrifugation did not increase the sensitivity of the Gram stain examination of AF. In that study, however, AF infection was frequently associated with high bacterial counts and the number of patients with low bacterial counts was small.

The purpose of this study was to evaluate the

**Address correspondence/reprint requests to Dr. Daniel W. Gauthier, Department of Obstetrics and Gynecology, Division of Maternal-Fetal Medicine, University of Illinois College of Medicine, 820 South Wood Street, M/C 808, Room 250, Chicago, IL 60612-7313.**

Received January 14, 1994

Accepted March 30, 1994

Basic Science Article effect of centrifugation on the sensitivity of AF Gram stain when performed with known concentrations of bacteria.

### SUBJECTS AND METHODS

AF was obtained by amniocentesis from patients between 24 and 34 weeks estimated gestational age with premature rupture of membranes (PROM) or preterm labor (PTL). All of the individual AF samples had an initial Gram stain negative for white blood cells (WBCs) and microorganisms as well as negative aerobic, anaerobic, and *Mycoplasma* cultures. The individual AF samples were then stored at −20°C. When sufficient AF had been collected to perform the study, the stored samples were thawed, pooled, and kept at 37°C. Repeat Gram stain and cultures were also negative on the pooled AF sample.

Using pure bacterial cultures of *Escherichia coli, Listeria monocytogenes, Neisseria gonorrhoeae,* and *Streptococcus agalactiae,* we prepared a suspension corresponding to a 0.5 McFarland standard ($3 \times 10^8$ colony forming units [cfu]/ml) for each organism. This concentration was confirmed with spectrophotometric technique and colony counts using appropriate media for each organism. The bacterial suspensions of each organism were then diluted in the pooled AF to either $10^3$, $10^4$, $10^5$, or $10^6$ cfu/ml using calibrated inoculating loops.

Each AF sample was then divided into 2 equal aliquots (1 ml each) and 1 was centrifuged at 1,200*g* for 10 min. The AF supernatant was discarded and the cellular pellet was resuspended in 1 drop (0.03 ml) of sterile normal saline. Four Gram stain examinations were performed with commercial reagents in the standard fashion from each sample. Sixteen Gram stain examinations of both spun and unspun pooled AF without added bacteria were also performed to serve as negative controls.

The Gram stain examinations were interpreted by 2 medical technologists from the clinical microbiology laboratory who did not participate in performing the Gram stains and who were blinded to the number of AF samples containing bacteria, the bacterial morphotypes, and the bacterial concentration. The slides were presented to the technologists in random order. Each slide was interpreted by only 1 technician as is currently done in clinical situations at our institution. Fisher's exact test was used to compare the bacterial detection rate of centrifuged vs. uncentrifuged samples at each concentration.

**TABLE 1. Comparison of Gram stain results in centrifuged and uncentrifuged AF samples**

| | Gram stain | | | |
|---|---|---|---|---|
| | Uncentrifuged AF | | Centrifuged AF | |
| cfu/ml | Positive | Negative | Positive | Negative |
| 0 | 1 | 15 | 1 | 15 |
| $10^3$ | 2 | 14 | 10 | 6 |
| $10^4$ | 7 | 9 | 12 | 4 |
| $10^5$ | 13 | 3 | 16 | 0 |
| $10^6$ | 15 | 1 | 15 | 1 |

### RESULTS

A total of 160 Gram stains were performed and interpreted. Table 1 describes the Gram stain results of centrifuged and uncentrifuged AF for each bacterial concentration. Bacteria were detected significantly more often in centrifuged AF when the bacterial concentration was $10^3$ cfu/ml ($P < 0.01$). At the bacterial concentration of $10^4$ cfu/ml, the difference between spun and unspun AF approached but did not reach significance ($P = 0.08$). When AF samples with different bacterial concentrations were combined, bacteria were detected significantly more often in centrifuged samples when the bacterial concentration was $\leq 10^4$ cfu/ml ($P < 0.01$). There was no significant difference in the ability of the Gram stain to detect bacteria with the use of centrifugation when the bacterial concentration was $\geq 10^5$ cfu/ml.

The ability to correctly identify the bacterial shape (rod vs. cocci) or color (gram positive vs. gram negative) was not affected by centrifugation. When we compared uncentrifuged AF with centrifuged AF, the shape and the color of the bacteria were correctly identified in 94% (35 of 37) vs. 98% (52 of 53) and 81% (30 of 37) vs. 85% (45 of 53), respectively.

### DISCUSSION

In patients with PTL or PROM who undergo amniocentesis to assess for intraamniotic infection, preparation of the AF prior to Gram stain has not been well studied. Centrifugations of the AF prior to Gram stain should increase the concentration of bacteria and increase the ability of the test to detect infection. In the present study, we found that at bacterial concentrations $\leq 10^4$ cfu/ml, centrifugation of the AF prior to Gram stain did increase its ability to detect bacteria. This improved detection rate was not demonstrated at higher bacterial concentrations because of the already high detection rate of the Gram stain in the unspun samples.

The findings of the present study are consistent with those of Romero et al.[4] The ability of the unspun Gram stain to detect bacteria according to bacterial concentration was similar when comparing their results with ours, with detection rates of 20% vs. 12.5%, 38% vs. 44%, and 81% vs. 81% at bacterial concentrations of $10^3$, $10^4$, and $\geq 10^5$ cfu/ml, respectively. However, Romero et al.[4] could not demonstrate a difference in the ability of the Gram stain to detect bacteria in centrifuged vs. uncentrifuged AF. A potential explanation given was that intraamniotic infection in their study was frequently associated with high bacterial counts and that an appropriate number of samples in the critical range necessary to prove a difference was not obtained. In their study, 21 AF samples (55%) analyzed quantitatively had more than $10^5$ cfu/ml, whereas only 9 samples (24%) had bacterial counts $\leq 10^4$ cfu/ml. We also could not demonstrate a difference in the ability of the Gram stain to detect bacteria in centrifuged vs. uncentrifuged AF when the bacterial concentration was $\geq 10^5$ cfu/ml.

In the current study, we used an in vitro approach with pooled AF with a known negative Gram stain and cultures. To this AF we added a confirmed specific amount of bacterium. We decided to perform the study in this fashion for 2 reasons. The first was to avoid the possibility of having a small number of AF samples with low bacterial counts. The second was to be assured that our assessment of bacterial concentration was accurate since there is evidence that AF may contain several potent antibacterial factors.[5] These antibacterial factors may affect accuracy when determining the bacterial concentration in infected AF.

The in vitro study design differs in 2 major ways from what is seen clinically. First, in our study, the AF was inoculated with pure samples of only 1 bacteria. However, at our institution, over 50% of patients with preterm PROM with positive AF cultures have a polymicrobial infection.[2] This should not affect the ability of the Gram stain to detect bacteria but may decrease the accuracy of correctly identifying bacterial shape and color. Second, WBCs are present in many AF samples from patients with positive cultures. Our study design excluded AF samples if WBCs were present. WBCs, especially in centrifuged AF, may be in great enough concentration to obscure the Gram stain and affect the ability to detect bacteria.

There were 2 false positive Gram stains noted in the study when no bacteria were added to the AF, 1 each in the centrifuged and uncentrifuged samples. Although the reason for the false results are not clearly obvious, the false positive Gram stains may be secondary to the cellular debris found in the AF. AF most likely contains more particulate matter than other body fluids (urine, cerebrospinal fluid) that are commonly centrifuged before Gram stain. When the AF was stored and then subsequently pooled, there was no attempt to remove vernix or other particulate matter since this is not done in clinical situations at our institution.

Centrifugation of the AF prior to analysis may improve the ability of the Gram stain to detect intraamniotic infection at bacterial concentrations $\leq 10^4$ cfu/ml. Further research is needed to assure that the increased bacterial detection rate obtained by centrifugation is not associated with a concurrent increase in false positive results. This could lead to an unnecessary delivery of a preterm infant if clinical management is based on Gram stain results alone.

### REFERENCES

1. Garite TJ, Freeman RK, Linzey M, Braly P: The use of amniocentesis in patients with premature rupture of membranes. Obstet Gynecol 54:226–230, 1979.
2. Gauthier DW, Meyer WJ: Comparison of Gram stain, leukocyte esterase activity and amniotic fluid glucose concentration in predicting amniotic fluid cultures in preterm premature rupture of membranes. Am J Obstet Gynecol 167:1092–1095, 1992.
3. Cotton DB, Hill LM, Strassner HT, Platt LD, Ledger WJ: Use of amniocentesis in preterm gestation with ruptured membranes. Obstet Gynecol 63:38–43, 1984.
4. Romero R, Emamian M, Quintero R, et al.: The value and limitations of the Gram stain examination in the diagnosis of intraamniotic infection. Am J Obstet Gynecol 159: 114–119, 1988.
5. Ledger WJ: Infection in the Female. 2nd Ed. Philadelphia: Lea & Febiger, pp 66–67, 1986.
